# Structural and nutritional portrayal of rye‐supplemented bread using fourier transform infrared spectroscopy and scanning electron microscopy

**DOI:** 10.1002/fsn3.2592

**Published:** 2021-09-20

**Authors:** Ali Ikram, Farhan Saeed, Muhammad Umair Arshad, Muhammad Afzaal, Faqir Muhammad Anjum

**Affiliations:** ^1^ Department of Food Science Government College University Faisalabad Pakistan; ^2^ University of the Gambia Serekunda Gambia

**Keywords:** FTIR, GC‐FID, rye bread, SEM

## Abstract

In the present study, four different variants, namely Gp‐1, Gp‐2, Gp‐3, and Gp‐4, were characterized for their nutritional and fatty acid profile. Later on, the nutritionally superior variant was used for bread preparation. Purposely, composite flour was prepared with different ratios of wheat and rye (100:0; 90:10; 80:20). Furthermore, structural characterization of bread was done using Fourier transform infrared (FTIR) spectroscopy and scanning electron microscopy (SEM). Results showed that the Gp‐2 was more nutritional among the four variants. Furthermore, the spectra of composite flour bread were scanned in the range of 4000–600 cm^–1^. All the bread samples presented almost similar spectra for major peaks corresponding to wavenumbers in the functional group. The SEM micrographs showed the presence of small and large starch particles with compact structures. Conclusively, rye flour supplementation has a significant impact on the nutritional and structural attributes of the bread.

## Graphical abstract

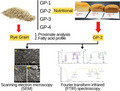

## 1 INTRODUCTION

Cereals are edible grains belonging to the *Gramineae* family. Rye, millet, barley, oats, triticale, maize, and sorghum are some grains grown in many countries. Wheat and rice are the most significant crops in the world, accounting for more than half of all cereal production. All cereals have an embryo (or germ) that holds the genetic material for a new plant and an endosperm that contains starch grains. It is genetically linked to barley and wheat (both belonging to the triticale family) (Grabiński et al., 2021). Rye, on the other hand, is tougher than barley and wheat. Compared to wheat, rye takes this into account with less than 1% of total worldwide cereal production. Worldwide 3% of the rye is produced as compared to other cereals (Sluková et al., 2021). The current global rye harvest is estimated to be around 14.8 million tons, with the majority of the crop grown in the northern half of the region, from the Nordic Sea to the Ural Mountains. Rye is exceptionally hardy in the winter and can be grown in sandy, low‐fertility soils, allowing it to be grown in places where other cereal crops are not appropriate. Poland, Russian Federation, Belarus, Germany, as well as Ukraine are the top rye producers. Over a quarter of the entire rye crop is consumed as food, primarily in bread. In the “Rye Belt” countries, rye consumption ranges from 10 to 30 kg per person/year (Deleu et al., 2020).

The rye flour covers protein 8%–13%, starch 56%–70%, ash 2%, lipids 2%–3%, and 15%–21% total dietary fiber. In the rye flour, higher levels of dietary fiber (15%–21%) are present compared with wheat (11%–13%). Arabinoxylan (AX; 8%–12%), cellulose (1%–1.7%), and beta‐glucan (1.3%–2.2%) are the main fibers present in the rye grains (Hansen et al., 2003). Fructans (including fructooligosaccharides) are also present in rye flour in the range of 4.6%–6.6% (Karppinen et al., 2003). Fructans are considered as a part of dietary fiber. The germ of the rye grains is rich in lipids, which are made up of unsaturated fatty acids. Oleic, linolenic, and linoleic acids found almost 81.6% of total fatty acids in the germ of rye grain (Ross et al., 2001).

The rye and wheat composite flours at different extraction rates are used for the preparation of rye bread. The dietary fiber content in rye bread is in the range of 6%–16% (Grabiński et al., 2021). Rye grain can offer diversity to the cereal diets available to humans while also increasing the levels of DF and other bioactive chemicals. As customer perception of rye and whole‐grain foods' health benefits increases, the cereal food sector is being challenged to offer novel nutritious rye products with better and various sensory profiles (Miedaner and Geiger, 2015).

Rye is a good source of phenolics, dietary fiber, minerals, and vitamins and is commonly eaten as whole grain products. Rye bread has been found to offer physiological benefits, particularly in terms of glucose metabolism and satiation. Despite significant variations in chemical and technological properties, rye (*Secale cereale, L.)* is very close to wheat among cereal grains. The husk on both grains is generally removed by threshing. The pericarp and testa, which contain the germ and endosperm (aleurone and starchy endosperm), are the outermost layers of the bare kernel (Shewry and Bechtel, 2001).

As a result, rye is a major source of dietary fiber (DF) in many countries. Sour and dark bread, crisp bread, flakes for porridges, loaf bread with sifted rye flour, and breakfast cereals are the most common rye dishes (Ghiafeh Davoodi et al., 2021). Owing to the nutritional profile of the rye flour, the present study was planned to explore the rye flour supplementation on different attributes of bread.

## 2 METHODOLOGY

### 2.1 Procurement of raw material

Rye grains were purchased from Forage Section, Ayub Agriculture Research Institute, Faisalabad. Four different gene pools named as RJS‐10001, RJS‐10002, RJS‐10003, and RJS‐10004 were coded as GP‐1, GP‐2, GP‐3, and GP‐4. All the chemicals required for the analysis were laboratory grade. H_2_SO_4_ and methanol were purchased from the Sigma‐Aldrich Chemical Co., whereas petroleum ether and n‐hexane were purchased from Merck. The seeds were cleaned physically to expel dirt, dust particles, seeds of different harvests, and outside matter.

### 2.2 Milling

Hammer‐type laboratory mill 120 perton was used for the milling of rye grains at Ayub Agriculture Research Institute Faisalabad, Pakistan. Sieves of 0.5–2.0 mm are used for the milling of rye grains.

### 2.3 Analysis of rye flour

#### 2.3.1 Proximate composition

All variants of rye flour samples analyzed for moisture, crude ash, crude protein, crude fiber, crude fat, and NFE were determined through AACC (2000) methods.

#### 2.3.2 Fatty acid profile

The fatty acid profile of rye was determined by GC‐FID as described by Medeiros and Simoneit (2007). Briefly, methyl ester was prepared by using the 2 g oil sample in 5 ml methanolic H_2_SO_4_ (40:10). Afterward, these were heated at 80°C for 2 h. After cooling the samples at room temperature, samples were transferred into test tubes and 1 ml of petroleum ether was added thrice in each test tube. Two milliliters of distilled water was added to the test tube and mixed by using a vortex mixer. For the complete formation of layers, 5–10 min stay time was given to the samples. The upper layer was removed and run in GC‐FID. For further analyses, flame ionization detector, Agilent DB‐5 with methyl polysiloxane polymer phase column, and nitrogen gas (N) as a carrier gas at a flow rate of 1.3 ml/min were used.

### 2.4 Blended flour and dough development

Among four variants, the nutritionally superior rye variant was selected for the preparation of composite flour and later on for bread preparation (Table 1).

**TABLE 1:** Treatment plan

Treatment	Wheat	Rye
T0	100%	–
T1	90%	10%
T2	80%	20%

### 2.5 Product development

#### 2.5.1 Preparation of bread

**FIGURE 1:**
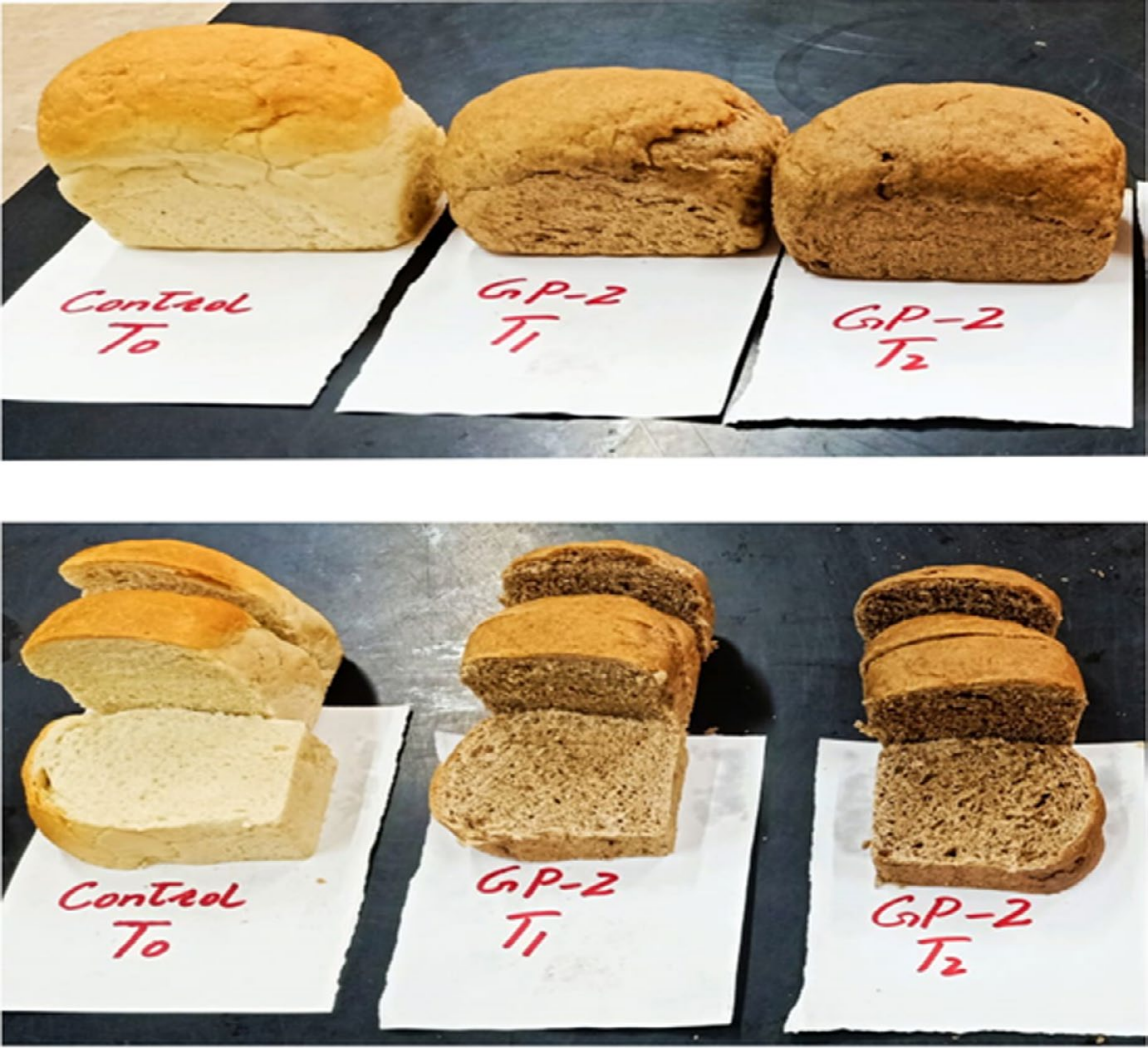
Bread prepared with supplemented flour

The ingredients, composite flour (rye+wheat), compressed fresh yeast, sugar, salt, improver, shortening, and water, were mixed with a mixer, and bread was prepared with a straight dough method at Ayub Agriculture Research Institute Faisalabad, Pakistan, using method no. 10–10.03 AACC (2000) (Figure 1).

### 2.6 Bread analysis

The bread samples were analyzed for the physicochemical analysis, i.e., bread loaf volume, water activity, moisture, crude protein, ash, crude fat, crude fiber, and NFE content, through AACC (2000) methods.

### 2.7 Structural characterization

The bread was structurally characterized using the Fourier transform infrared spectroscopy (FTIR) and scanning electron microscopy (SEM).

#### 2.7.1 Fourier transform infrared spectroscopy

As stated in the Instruction Manual IR Prestige‐21, functional components of each sample were identified using FTIR. The Shimadzu Fourier transform infrared spectrophotometer‐FTIR 8400 S was used to determine functional units. The scanned sample traveled through infrared, where it was detected as a continuous wave by a computer‐connected detector and the spectrum of the tested sample was reported. Typically, samples were scanned in the absorbance range of 600–4000 cm‐1. As the basis of spectrum type, the findings of the investigation included the chemical structure, molecular binding form, and specific functional groups of the examined material (Sivam et al., 2013).

#### 2.7.2 Scanning electron microscopy

The bread was sputter‐coated using gold in a sputter coater after being mounted on a stub with double‐sided sticky tape (JEC‐3000FC). A scanning electron microscope (cube series company, Emcraft) was used to image the samples (800) at a 5‐kV accelerating voltage. The images were recorded as previously described by (Wang et al., 2017 **)**.

### 2.8 Statistical analysis

The resultant data were statistically analyzed by CRD using Minitab statistical package described by (Steel, 1997). The results were presented as mean values ± standard deviation (*SD*).

## 3 RESULTS AND DISCUSSION

### 3.1 Proximate analysis of rye

The results regarding the proximate analysis of rye are shown in Table 2. Results showed that the moisture content in the different rye flours ranged from 7.01% to 7.6%. The maximum moisture content (7.6%) was present in the GP‐2, whereas the lowest (7.01%) was observed in the GP‐1. The ash content ranged from 1.47% to 1.97% in the different rye flours. The maximum ash content (1.97%) was observed in Gp‐3, whereas the lowest ash content (1.47%) was observed in the Gp‐1.

**TABLE 2:** Proximate analysis of rye flour

	Proximate analysis (%)						Fatty acid profile (%)							
	Moisture	Ash	Crude protein	Crude fat	Crude fiber	NFE	Myristic acid (14:0)	Palmitic acid (16:0)	Oleic acid (18:1)	Linoleic acid (18:2)	Arachidic acid (20:0)	Behenic acid (22:0)	EPA (20:5)	DHA (22:6)
Gp−1	7.01 ± 0.37	1.47 ± 0.35	9.13 ± 0.65	1.76 ± 0.47	2.5 ± 0.4	78.13 ± 1.71	0.87 ± 0.02	12.34 ± 0.02	3.31 ± 0.18	7.95 ± 0.01	2.63 ± 0.02	1.05 ± 0.015	3.95 ± 0.02	0.87 ± 0.01
GP−2	7.6 ± 0.2	1.8 ± 0.45	9.2 ± 0.75	1.86 ± 0.35	2.8 ± 0.36	76.74 ± 0.75	0.90 ± 0.02	11.30 ± 0.015	4.63 ± 0.15	7.73 ± 0.15	2.67 ± 0.02	2.09 ± 0.07	4.01 ± 0.08	0.88 ± 0.04
Gp−3	7.54 ± 0.35	1.97 ± 0.4	9.56 ± 0.64	1.8 ± 0.56	2.67 ± 0.35	76.5 ± 0.76	0.86 ± 0.01	12.35 ± 0.15	6.96 ± 0.15	7.62 ± 0.01	2.61 ± 0.015	0.08 ± 0.03	3.97 ± 0.015	0.80 ± 0.05
Gp−4	7.58 ± 0.4	1.67 ± 0.25	9.4 ± 0.6	1.73 ± 0.25	2.74 ± 0.62	76.96 ± 0.71	0.85 ± 0.02	12.21 ± 0.02	5.5 ± 0.2	7.84 ± 0.02	2.56 ± 0.02	1.04 ± 0.004	3.89 ± 0.02	0.83 ± 0.02

The crude protein content in the various rye content ranged from 9.13% to 9.56%. Gp‐3 showed the maximum protein content (9.56%); meanwhile, minimum protein content (9.13%) was noticed in the Gp‐1. The crude fat content ranged from 1.73% to 1.86% in the different rye flours. The maximum crude fat content (1.86%) was noticed in the Gp‐2, whereas the lowest (1.73%) was observed in the Gp‐4. The crude fiber in the rye flour ranged from 2.5% to 2.8%. The maximum crude fiber content (2.8%) was present in the Gp‐2, while the lowest (2.5%) was noticed in the Gp‐1. The nitrogen‐free extract (NFE) in the rye flours ranged from 76.5% to 78.13%. The maximum NFE (78.13%) was observed in the Gp‐1, whereas the lowest amount (76.5%) was presented in the Gp‐3. These results are in accordance with the finding of Drakos et al. (2017) who investigated the effect of jet milling physicochemical and mechanical properties of rye and barley flours. Another study conducted by Cardoso et al. (2019) found that the moisture, ash, protein, fat, and NFE were 9.3, 1.27, 8.2, 1.31, and 89.2 g/100g.

### 3.2 Fatty acid profile of rye flour

The results concerning the fatty acid content of rye are shown in Table 2. The results showed that the myristic acid was present in the range of 0.85%–0.90%. The maximum amount of myristic acid (0.90%) was observed in the Gp‐2, while the minimum content (0.85%) was observed in Gp‐4. The palmitic acid was observed in the range of 11.30%–12.35% throughout the samples. The maximum palmitic acid content (12.35%) was noticed in the Gp‐3, whereas the minimum palmitic acid (11.30%) was observed in the Gp‐2. The oleic acid content in the rye flour was observed in the range of 3.31%–6.96%. The maximum oleic acid content (3.31%) was noticed in the Gp‐2, while the minimum oleic acid (6.96%) was observed in the Gp‐3. The linoleic acid content was present in the range of 47.92%–47.95%. The maximum linoleic acid content (47.95%) was observed in the Gp‐1, while the lowest (47.92%) was noticed in Gp‐3. The rye flour showed that the arachidic acid was present in the range of 2.56%–2.67%. The highest amount of arachidic acid (2.67%) was present in the Gp‐2, while the lowest amount of arachidic acid (2.56%) was observed in the Gp‐4. Behenic acid is one of the fatty acids, which is present in the range of 0.08%–2.09%. Behenic acid (2.09%) was present maximum in the Gp‐2, and meanwhile, minimum (0.08%) was observed in the Gp‐4. The results regarding the EPA (eicosapentaenoic acid) showed that in the rye flour EPA was noticed in the range of 3.89%–4.01%. The maximum EPA content (4.01%) was presented in the Gp‐2, whereas minimum EPA content (3.89%) was noticed in the Gp‐2. Docosahexaenoic acid (DHA) in the rye flour was present in the range of 0.80%–0.88%. The maximum DHA content (0.88%) was present in the Gp‐2, whereas minimum DHA content (0.80%) was noticed in the Gp‐3. The results are in accordance with the finding of Phillips et al. (2020) who found that the myristic (C14:0), palmitic, oleic (C18:1), oleic acid (C16:0), linoleic (C18:2) acid, arachidic (C20:0) acid, and behenic (C22:0) acid were 0.9, 17.3, 2, 8.2, 2.3, and 1/100 g, respectively. Similar results were also found by Bağcı et al. (2019) during the study of the mineral and fatty acid composition of rye grains. Another study conducted by Phillips et al. (2020) presented similar findings.

### 3.3 Bread analysis

The results regarding the physicochemical analysis of the composite bread are shown in Table 3. Results showed that the bread loaf volume of the bread was ranged from 231.3 to 236.31 cm^3^. The control bread (T0) showed the maximum bread loaf volume (236.31 cm^3^), whereas the T2 (231.3 cm^3^) presented the lowest bread loaf volume. As the rye flour in the bread increases, the loaf volume decreases just because of the high fiber content in the rye flour. The water activity of the bread ranged from 0.932 to 0.945. The maximum water activity of the bread was noticed in the T2 (0.945), whereas T1 (0.932) showed the lowest water activity. Bread's water activity reveals the microbiological growth's lower limit in terms of water availability. The moisture content of the bread ranged from 35.3% to 41.2%. The maximum moisture content (41.2%) was present in the T2, while the lowest moisture content (35.3%) was noticed in the T0. The ash content in the bread ranged from 0.8% to 1.2%. The maximum ash content (1.2%) was present in the T2, whereas minimum ash content (0.8%) was observed in the T1. The crude protein in the bread ranged from 9.7% to 11.6%. The maximum crude protein content (11.6%) was present in the T2; meanwhile, the lowest (9.7%) was noticed in the T0. The crude fat in all the samples of bread ranged from 3.3% to 4.2%. The maximum crude fat content (4.2%) was present in the T2, whereas minimum crude fat content (3.3%) was observed in the T0. The crude fiber content of the bread ranged from 2.9% to 8.1%. The T2 bread sample showed the highest amount of crude fiber (8.1%), whereas the lowest crude fiber content was (2.9%) in T0. The nitrogen‐free extract (NFE) ranged from 74% to 83% in the bread samples. The maximum NFE content (83%) was shown in the T0, whereas the lowest content NFE was (74%) in T2. The results are in accordance with the findings of Sankhon et al. (2013).

**TABLE 3:** Physicochemical analysis of rye bread

	Bread loaf volume (cm^3^)	Water activity	Moisture (%)	Ash (%)	Crude protein (%)	Crude fat (%)	Crude fiber (%)	NFE
To	236.31 ± 2.8	0.941 ± 0.3	35.3 ± 0.5	1.1 ± 0.9	9.7 ± 0.8	3.3 ± 0.8	2.9 ± 0.9	83 ± 0.6
T1	234.17 ± 1.4	0.932 ± 0.5	39.8 ± 1.2	0.8 ± 0.7	10.2 ± 0.5	4.1 ± 0.5	6.5 ± 1.3	77.9 ± 0.4
T2	231.3 ± 0.5	0.945 ± 1.2	41.2 ± 0.8	1.2 ± 0.6	11.6 ± 0.6	4.2 ± 1.2	8.1 ± 1.2	74 ± 0.5

### 3.4 Structural characterization of bread

#### 3.4.1 Fourier transform infrared analysis

**FIGURE 2:**
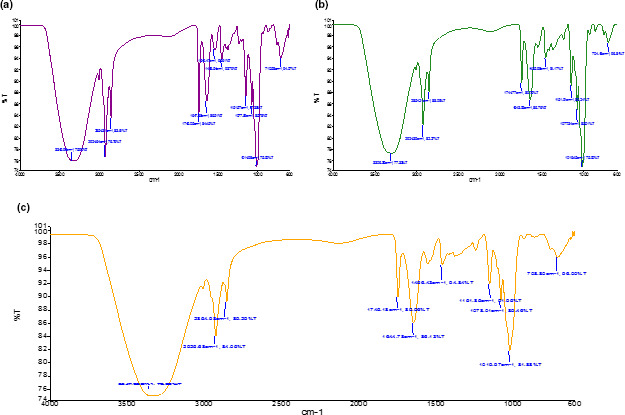
(a) Fourier transform infrared spectroscopic (FTIR) of T0 (control). (b) Fourier transform infrared spectroscopic (FTIR) of T1 (90:10). (c) Fourier transform infrared spectroscopic (FTIR) of T2 (80:20)

The Fourier transform infrared (FTIR) spectroscopic analysis was conducted to support the proximate data for possible variation in the composition of major fractions such as moisture, proteins, fats, and saccharides of composite bread (Figure 2). The spectra of wheat flour and rye composite bread were scanned in the range of 4000–600 cm^–1^, and almost 11 nominal peaks corresponding to the constituents were evaluated. All the samples of bread presented almost similar spectra for major peaks corresponding to wavenumbers in the functional group region (4000–1200 cm^–1^) and the unique pattern also called fingerprint region (1200–600 cm^–1^) (Cocchi et al., 2004; Guo et al., 2015; Sujka et al., 2017). However, small yet significant changes were noticed in the spectra where the amplitude of few peaks was modified by replacing the wheat flour with rye flour. The major broad peak articulated around 3350–3300 cm^–1^ indicates the stretching vibrations of inter‐ or intramolecular O–H bonds, which showed a minor redshift in the spectra of composite flour, suggesting changes in the sample moisture and possible interactions between water and starch or proteins.

The narrow sharp but small peaks centered at 2924 and 2854 cm^–1^ majorly contributed by the symmetric stretching vibrations of the C–H bonds of the –CH_2_/–CH_3_ alkyl groups. These peaks are reportedly suggesting the presence of lipids mostly bound ones (Su & sun, 2018). The increased transmittance of these peaks as seen by reduced amplitude dictates the reduction of lipid fractions in the presence of rye in the composite bread flours. The narrow peak centered at 1745 cm^–1^ corresponds to the presence of C=O stretching vibrations. The most interesting peaks related to protein amide groups are found in the region of 1700–1200 cm^–1^. Mainly, two peaks at around 1647 and 1549 cm^–1^ were noticed in all the bread flour samples with or without rye, where the former corresponds to the stretching vibration for the C=O of the amide–I, while the later peak denotes the C–N stretching coupled with N–H bending of the amide–II. The phase composition and interface of the starch gelatin blend were studied by synchrotron FTIR micro‐spectroscopy. Linlaud et al. (2011) suggested the presence of gluten secondary structure as α‐helical at a peak around 1650 cm^–1^. However, no much change in the peak amplitude and symmetry was noticed for all the bread flour samples. However, amide–III has corresponded with the presence of a mild peak in the range of 1280–1220 cm^–1^, resulting from the β‐sheets of gluten. This peak is also allocated to the combined bending and stretching vibrations of N–H and C–H. Similarly, 1450 cm^–1^ peak in the bread flour samples is related to the C–H bending of the proteins. Again, there was not much change in the peak intensity between the control wheat bread and the composite samples.

The wavenumber ranging from 1200 to 800 cm^–1^ is assigned to the presence of polysaccharides in the bread flour. The intense peak at 1014 cm^−1^ is originated from the stretching of C–O and twisting vibrations of CH_2_ in the –CH_2_OH units (Wang & Somasundaran, 2007). A shoulder peak of less intensity was also noticed at 1077 cm^−1^ in all the samples, attributed to the C–O–H bending vibrations of the glycosidic linkages. Similarly, another shoulder at 1151 cm^−1^ has been correlated with the C–H stretching of the starch (Mathlouthi & Koenig, 1987). Interestingly, these peaks between 1200–800 cm^−1^ shifted to a higher transmittance for the composite bread compared to the control wheat bread suggesting the possible changes in the configuration of starch and other high molecular weight fractions of the flour. Similar, functional and molecular skeleton and fingerprinting regions were reported previously when wheat flour was mixed with barley and other ingredients.

#### 3.4.2 Scanning electron microscopy

**FIGURE 3:**
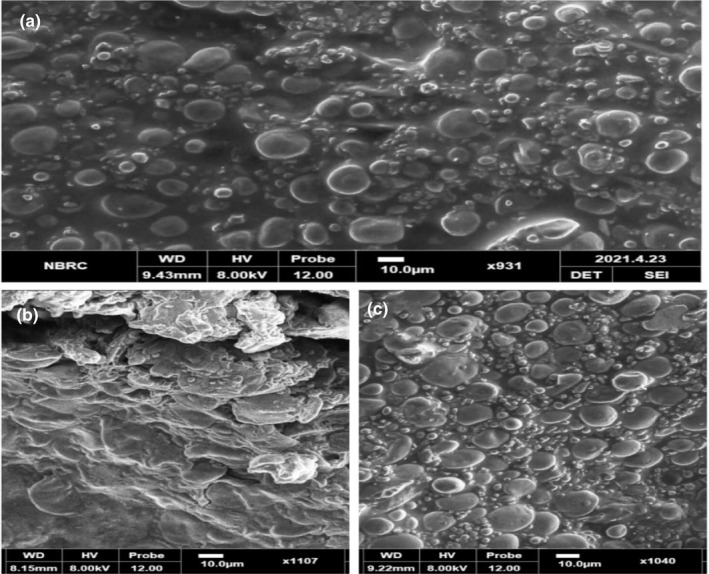
(a) Scanning electron microscopic diagram of T0 (control) bread. (b) Scanning electron microscopic diagram of T1 (90:10) bread. (c) Scanning electron microscopic diagram of T2 (80:20) bread

Scanning electron microscopy studies of all the samples were done to characterize the bread structure. Scanning electron micrographs of prepared bread samples are shown in Figure 3. The micrographs of control bread showed the presence of small and large starch particles. The gluten‐developed structure can be seen in the micrographs of the control bread sample. Likewise, the sample prepared with rye flour supplementation showing a more compact structure and low bread volume compared to the control. Furthermore, the addition of 20% flour supplementation showed a more compact structure. The results of the study are in line with other researchers who proposed that the micrographs of control bread varied from treated bread samples. Similarly, in another study, it was reported that dough treated with enzymes gave different micrographs compared to control. Micrographs of the study showed that starch particles were embedded in gluten structure (Ahlborn et al., 2005). The presence of supplemented rye flour exhibited a significant effect on the micrographs of different bread compared to control.

## 4 CONCLUSION

In the four different variants of the rye, Gp‐2 was found to be more nutritionally superior. Rye flour supplementation has a significant impact on the nutritional and structural attributes of the bread. Furthermore, the bread prepared with rye flour supplementation (80:20) shows a more compact structure and low bread volume compared to control. The presence of supplemented rye flour exhibited a significant effect on the micrographs of different bread compared to control. Rye flour supplementation should be used to improve the technological and functional properties of baked products.

## CONFLICT OF INTEREST

The authors declare that they do not have any conflict of interest.

## ETHICAL APPROVAL

This study does not involve any human or animal testing.

## STUDIES INVOLVING HUMAN SUBJECTS

This study does not involve any human testing.

## STUDIES INVOLVING ANIMAL OR HUMAN SUBJECTS

This study does not involve any human or animal testing.

## References

[fsn32592-bib-0001] Ahlborn, G. J. , Pike, O. A. , Hendrix, S. B. , Hess, W. M. , & Huber, C. S. (2005). Sensory, mechanical, and microscopic evaluation of staling in low‐protein and gluten‐free breads. Cereal Chemistry Journal, 82(3), 328–335. 10.1094/CC-82-0328

[fsn32592-bib-0002] Bağcı, A. , Gecgel, Ü. , Dursun, N. , Özcan, M. M. , Tamkoç, A. , Özer, İ. , & Özcan, M. M. (2019). The oil yields, mineral contents and fatty acid compositions of some rye (*Secale cereale*) grains. Iranian Journal of Chemistry and Chemical Engineering (IJCCE), 38(5), 285–292.

[fsn32592-bib-0003] Cardoso, R. V. , Fernandes, Â. , Heleno, S. A. , Rodrigues, P. , Gonzaléz‐Paramás, A. M. , Barros, L. , & Ferreira, I. C. (2019). Physicochemical characterization and microbiology of wheat and rye flours. Food Chemistry, 280, 123–129. 10.1016/j.foodchem.2018.12.063 30642477

[fsn32592-bib-0004] AACC . (2000). Approved methods of the AACC. American Association of Cereal Chemists.

[fsn32592-bib-0005] Cocchi, M. , Foca, G. , Lucisano, M. , Marchetti, A. , Pagani, M. A. , Tassi, L. , & Ulrici, A. (2004). Classification of cereal flours by chemometric analysis of MIR spectra. Journal of Agricultural and Food Chemistry, 52(5), 1062–1067. 10.1021/jf034441o 14995098

[fsn32592-bib-0006] Deleu, L. J. , Lemmens, E. , Redant, L. , & Delcour, J. A. (2020). The major constituents of rye (*Secale cereale* L.) flour and their role in the production of rye bread, a food product to which a multitude of health aspects are ascribed. Cereal Chemistry, 97(4), 739–754. 10.1002/cche.10306

[fsn32592-bib-0007] Drakos, A. , Kyriakakis, G. , Evageliou, V. , Protonotariou, S. , Mandala, I. , & Ritzoulis, C. (2017). Influence of jet milling and particle size on the composition, physicochemical and mechanical properties of barley and rye flours. Food Chemistry, 215, 326–332. 10.1016/j.foodchem.2016.07.169 27542482

[fsn32592-bib-0008] Ghiafeh Davoodi, M. , Karimi, M. , Naghipour, F. , & Sheikholeslami, Z. (2021). Investigating on increasing the shelf life of steamed bread containing rye flour by adding polyol and modifying the fermentation. Food Science and Technology, 18(115), 299–309.

[fsn32592-bib-0009] Grabiński, J. , Sułek, A. , Wyzińska, M. , Stuper‐Szablewska, K. , Cacak‐Pietrzak, G. , Nieróbca, A. , & Dziki, D. (2021). Impact of genotype, weather conditions and production technology on the quantitative profile of anti‐nutritive compounds in rye grains. Agronomy, 11(1), 151.

[fsn32592-bib-0010] Guo, X. X. , Hu, W. , Liu, Y. , Gu, D. C. , Sun, S. Q. , Xu, C. H. , & Wang, X. C. (2015). Rapid analysis and quantification of fluorescent brighteners in wheat flour by tri‐step infrared spectroscopy and computer vision technology. Journal of Molecular Structure, 1099, 393–398. 10.1016/j.molstruc.2015.06.081

[fsn32592-bib-0011] Hansen, H. B. , Rasmussen, C. V. , Bach Knudsen, K. E. , & Hansen, Å. (2003). Effects of genotype and harvest year on content and composition of dietary fibre in rye (*Secale cereale* L) grain. Journal of the Science of Food and Agriculture, 83(1), 76–85. 10.1002/jsfa.1284

[fsn32592-bib-0012] Karppinen, S. , Myllymäki, O. , Forssell, P. , & Poutanen, K. (2003). Fructan content of rye and rye products. Cereal Chemistry, 80(2), 168–171. 10.1094/CCHEM.2003.80.2.168

[fsn32592-bib-0013] Linlaud, N. , Ferrer, E. , Puppo, M. C. , & Ferrero, C. (2011). Hydrocolloid interaction with water, protein, and starch in wheat dough. Journal of Agricultural and Food Chemistry, 59(2), 713–719. 10.1021/jf1026197 21175189

[fsn32592-bib-0014] Mathlouthi, M. , & Koenig, J. L. (1987). Vibrational spectra of carbohydrates. Advances in Carbohydrate Chemistry and Biochemistry, 44, 7–89. 10.1016/S0065-2318(08)60077-3 3544701

[fsn32592-bib-0015] Medeiros, P. M. , & Simoneit, B. R. (2007). Analysis of sugars in environmental samples by gas chromatography–mass spectrometry. Journal of Chromatography A, 1141(2), 271–278. 10.1016/j.chroma.2006.12.017 17207493

[fsn32592-bib-0016] Miedaner, T. , & Geiger, H. H. (2015). Biology, genetics, and management of ergot (*Claviceps* spp.) in rye, sorghum, and pearl millet. Toxins, 7(3), 659–678. 10.3390/toxins7030659 25723323 PMC4379517

[fsn32592-bib-0018] Phillips, H. N. , Heins, B. J. , Delate, K. , & Turnbull, R. (2020). Fatty acid composition dynamics of rye (*Secale cereale* L.) and wheat (*Triticum aestivum* L.) forages under cattle grazing. Agronomy, 10(6), 813.

[fsn32592-bib-0019] Ross, A. B. , Kamal‐Eldin, A. , Jung, C. , Shepherd, M. J. , & Åman, P. (2001). Gas chromatographic analysis of alkylresorcinols in rye (*Secale cereale* L) grains. Journal of the Science of Food and Agriculture, 81(14), 1405–1411. 10.1002/jsfa.956

[fsn32592-bib-0020] Sankhon, A. , Amadou, I. , & Yao, W. R. (2013). Application of resistant starch in bread: Processing, proximate composition and sensory quality of functional bread products from wheat flour and African locust bean (*Parkia biglobosa*) flour. Agricultural Sciences, 4(05), 122.

[fsn32592-bib-0021] Shewry, P. R. , & Bechtel, D. B. (2001). Morphology and chemistry of the rye grain. In Rye: Production, chemistry and technology. St Paul, MN: AACC International Inc.

[fsn32592-bib-0022] Sivam, A. S. , Sun‐Waterhouse, D. , Perera, C. O. , & Waterhouse, G. I. N. (2013). Application of FT‐IR and raman spectroscopy for the study of biopolymers in breads fortified with fibre and polyphenols. Food Research International, 50(2), 574–585. 10.1016/j.foodres.2011.03.039

[fsn32592-bib-0023] Sluková, M. , Jurkaninová, L. , Švec, I. , & Skřivan, P. (2021). Rye–the nutritional and technological evaluation in Czech cereal technology–a review: Grain and flours. Czech Journal of Food Sciences, 39(1), 3–8. 10.17221/203/2020-CJFS

[fsn32592-bib-0024] Steel, R. G. D. (1997). Analysis of variance II: Multiway classifications (pp. 204–252). In Principles and procedures of statistics: A biometrical approach.

[fsn32592-bib-0025] Su, W. H. , & Sun, D. W. (2018). Fourier transform infrared and Raman and hyperspectral imaging techniques for quality determinations of powdery foods: A review. Comprehensive Reviews in Food Science and Food Safety, 17(1), 104–122. 10.1111/1541-4337.12314 33350060

[fsn32592-bib-0026] Sujka, K. , Koczoń, P. , Ceglińska, A. , Reder, M. , & Ciemniewska‐Żytkiewicz, H. (2017). The application of FT‐IR spectroscopy for quality control of flours obtained from polish producers. Journal of Analytical Methods in Chemistry, 2017, 1–9. 10.1155/2017/4315678 PMC5294222 28243483

[fsn32592-bib-0027] Wang, J. , & Somasundaran, P. (2007). Study of galactomannose interaction with solids using AFM, IR and allied techniques. Journal of Colloid and Interface Science, 309(2), 373–383. 10.1016/j.jcis.2006.10.086 17316669

[fsn32592-bib-0028] Wang, S. , Yu, M. , Jiang, J. , Zhang, W. , Guo, X. , Chang, S. , Campbell, M. (2017). Evidence aggregation for answer re‐ranking in open‐domain question answering. arXiv preprint arXiv:1711.05116.

